# Autism risk classification using placental chorionic surface vascular network features

**DOI:** 10.1186/s12911-017-0564-8

**Published:** 2017-12-06

**Authors:** Jen-Mei Chang, Hui Zeng, Ruxu Han, Ya-Mei Chang, Ruchit Shah, Carolyn M. Salafia, Craig Newschaffer, Richard K. Miller, Philip Katzman, Jack Moye, Margaret Fallin, Cheryl K. Walker, Lisa Croen

**Affiliations:** 1 Department of Mathematics and Statistics, California State University, Long Beach, Long Beach, CA 90840-1001 USA; 2 Department of Statistics, Tamkang University, No.151, Yingzhuan Rd., New Taipei City, 25137 Taiwan; 3 Placental Analytics, LLC, New Rochelle, NY, USA; 4 Institute for Basic Research, Staten Island, NY, USA; 5 NIH National Children’s Study Placenta Consortium, Bethesda, MD, USA; 6 Drexel University, Philadelphia, PA, USA; 7 University of Rochester, Rochester, NY, USA; 8 NICHD, Bethesda, MD, USA; 9 Johns Hopkins University, Baltimore, MD, USA; 10 University of California Davis, Davis, CA, USA; 11 Kaiser Permenante Division of Research, Oakland, CA, USA

**Keywords:** Placental chorionic surface vascular network (PCSVN), Autism spectrum disorder risk, Boruta algorithm, Linear discriminant analysis, Placenta, Principal component analysis, Random forest, Arterial network

## Abstract

**Background:**

Autism Spectrum Disorder (ASD) is one of the fastest-growing developmental disorders in the United States. It was hypothesized that variations in the placental chorionic surface vascular network (PCSVN) structure may reflect both the overall effects of genetic and environmentally regulated variations in branching morphogenesis within the conceptus and the fetus’ vital organs. This paper provides sound evidences to support the study of ASD risks with PCSVN through a combination of feature-selection and classification algorithms.

**Methods:**

Twenty eight arterial and 8 shape-based PCSVN attributes from a high-risk ASD cohort of 89 placentas and a population-based cohort of 201 placentas were examined for ranked relevance using a modified version of the random forest algorithm, called the Boruta method. Principal component analysis (PCA) was applied to isolate principal effects of arterial growth on the fetal surface of the placenta. Linear discriminant analysis (LDA) with a 10-fold cross validation was performed to establish error statistics.

**Results:**

The Boruta method selected 15 arterial attributes as relevant, implying the difference in high and low ASD risk can be explained by the arterial features alone. The five principal features obtained through PCA, which accounted for about 88% of the data variability, indicated that PCSVNs associated with placentas of high-risk ASD pregnancies generally had fewer branch points, thicker and less tortuous arteries, better extension to the surface boundary, and smaller branch angles than their population-based counterparts.

**Conclusion:**

We developed a set of methods to explain major PCSVN differences between placentas associated with high risk ASD pregnancies and those selected from the general population. The research paradigm presented can be generalized to study connections between PCSVN features and other maternal and fetal outcomes such as gestational diabetes and hypertension.

## Background

Autism Spectrum Disorder (ASD) is a neurodevelopmental disorder with deficits in three defining areas: social reciprocity, communication, and restricted and repetitive patterns of behaviors. Symptoms are typically developed by 36 months of age. The causes of ASD are not definitive and include both genetic and non-inherited factors and exposures. About one in 68 children in the United States and one percent of the world population has been identified with ASD, according to a 2016 estimate from the Center for Disease Control [1]. The lifelong cost of ASD in the United States is about $2.4 million for a person with an intellectual disability, or $1.4 million for a person without intellectual disability [2]. Since the brain is most responsive to treatment in the first year of life, early intervention is key to help children diagnosed with ASD. However, since most of the diagnoses of ASD are not made until the child is three or four years old, the best opportunities for intervention have already been lost. There is no doubt that ASD is a global epidemic and efforts are needed in developing reliable bio-markers in assessing prenatal and neonatal risk to not only increase the effectiveness of the treatments and minimize the cost to treat children with ASD.

One way to develop a bio-marker is to study groups of children exposed to high risk for ASD. For example, children with a twin sibling have a much higher chance of getting diagnosed with ASD. In particular, studies have shown that among identical twins, if one child has ASD, then the other will be affected about 36–95% of the time. In non-identical twins, if one child has ASD, then the other is affected about 0–31% of the time [3, 4]. Moreover, parents who have a child with ASD have a 2–18% chance of having a second child who is also affected [5, 6]. Based on a research study completed by the Baby Siblings Research Consortium [7], the recurrence risk of ASD was 18.7% for families with at least one older sibling with ASD. Children with more than one older sibling with ASD were even more likely to be diagnosed, with a 32.2% risk – twice that of children with only one older autistic sibling [7].

As we know that the gene families that control branching morphogenesis in the permanent organs such as kidneys, lungs, and pancreas are related to the genes that control branching morphogenesis in placenta [8], this makes placenta an ideal organ to study fetal vasculogenesis and angiogenesis. Abnormal placental angiogenesis and vasculogenesis underly a number of pregnancy complications, from preeclampsia to fetal growth restriction and pre-term birth [9–11]. Evidence suggests that it may also be responsible for irregular placental shape [12, 13]. A major feature of the whole placenta, the placental chorionic surface vascular network (PCSVN), has not been extensively studied due to the extreme difficulty in reliably extracting PCSVN features from digital images of the fetal surface [14]. It was hypothesized that variation in PCSVN structure, the template of the fetal organ positioned at the interface of the mother and the conceptus, may reflect both the overall effects of genetic and/or environmentally regulated (e.g., [15]) variations in branching morphogenesis within the conceptus, and may also mirror vascular network alterations in the fetus’ vital organs.

Although there were results linking chorionic surface shapes to immediate neonatal outcomes such as birth weight after adjustment for gestational length [13, 16], very limited work has been done on the connection between PCSVN features and neonatal outcomes. Preliminary research results [17] suggested that there are significant differences in PCSVN features (e.g., Number of branch generations and angles of vascular branching.) in children at increased risk for autism spectrum disorder. The study was conducted on a data set of 109 placentas with 33 from a high-risk ASD cohort and 76 from a population-based cohort, and did not include a mechanism to classify a given placenta as high-risk for ASD with the PCSVN features that were deemed significant.

**Fig. 1:**
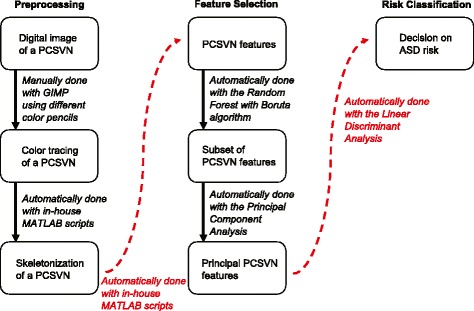
A flowchart of the research pipeline. The proposed work follows a three-stage process: preprocessing (left), feature-selection (middle), and classification (right). The entire process is automated except to obtain the color tracing

Our goal in this paper is to provide sound evidences to support the study of ASD risks through the placental chorionic surface vascular networks. We will do so by developing a set of methods to explain major PCSVN differences between placentas associated with high risk ASD pregnancies and those selected from the general population. The methods assume no a-priori knowledge on which factors might have been in play to establish the difference and can be generalized naturally to other maternal and fetal outcomes such as gestational diabetes and hypertension. A flowchart of our proposed work is given in Fig. 1. We will begin by describing the ways we obtain PCSVN features from a digital photograph of a placenta (preprocessing stage of Fig. 1), then discussing the methods we use to distill a relatively large set of PCSVN features into a subset of physically meaningful ones (feature selection stage of Fig. 1), and finally presenting the way the ASD risk is assigned (classification stage of Fig. 1).

## Methods

### Data sets

The placentas investigated in this study are taken from two independently collected cohorts, Early Autism Risk Longitudinal Investigation (EARLI) [18] and National Children’s Study (NCS). Protocols for the original data collections were approved by the pertinent Institutional Review Boards. This study concerns with secondary analysis on de-identified data.

EARLI is an autism enriched-risk pregnancy cohort that focuses on the prenatal and early life periods of children who have biological siblings already diagnosed with ASD. EARLI children are at an increased risk for ASD. On the other hand, NCS is a population-based cohort with pregnancies at unknown risk for ASD. NCS was designed to study environmental influences on child health and development and it enlisted participants without a bias towards risks and diagnoses in autism. Placentas in NCS are used here as an unselected low-risk baseline. We randomly selected 201 placentas from NCS and 89 placentas from EARLI in this study.

We have limited clinical data such as gender, gestation age, placental weight, and birth weights on small subsets of NCS and EARLI. Our data sets will be reduced significantly if we were to include these clinical attributes in the study; hence, the present study concerns only with the connections between vascular features of the PCSVN and risk outcomes for ASD.

### Vascular features

**Fig. 2:**
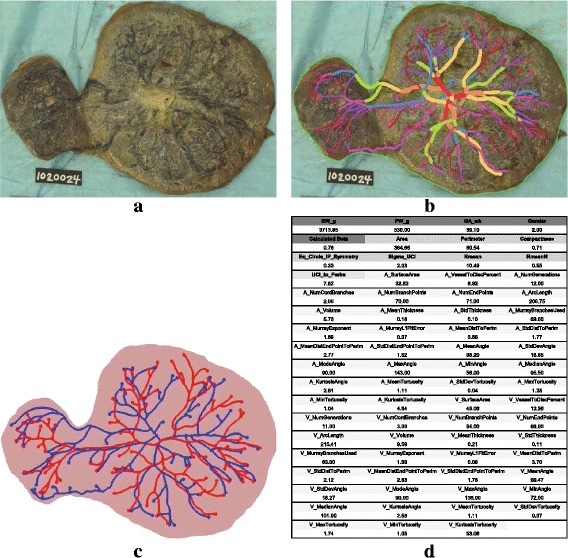
The process of obtaining a feature vector for each placenta. **a** A digital photograph of the placental chorionic surface vascular network (PCSVN) from the NCS data set. **b** Traced PCSVN for the image in (**a**) following the tracing protocols in [14]. **c** The skeletonisation of the traced PCSVN image in (**b**) that was produced by a MATLAB program written in house by the research team. **d** Numerical values of PCSVN features computed by our MATLAB program for the image in (**c**). Each of the 290 placentas in our data set is associated with a list of values similar to those given in (**d**)

Digital photographs of the fetal surface were obtained on 201 NCS placentas and 89 EARLI placentas following the same imaging protocol (e.g., Fig. 2 a). The photos of the placentas were taken either at delivery or upon pathology evaluation with fresh tissue. The raw PCSVN images in both NCS and EARLI data sets were captured using the same camera and polarizing filter. The distance between the camera and the placenta being imaged was fixed in NCS while there was a slight variability among EARLI images. Lighting condition was also fixed in NCS while there was a slight variability in lighting among EARLI images.

PCSVN of each placenta was first traced manually (e.g., Fig. 2 b) following the protocol documented in [14] using GIMP by one of the researchers who was blind to the risk categories. To make the manual tracing consistent and compatible with the computer algorithms, the researchers in [14] developed a protocol in which different colors and pencil sizes were used to mark different vessel thicknesses and separate the placental chorionic surface arterial network from the subjacent venous network. All tracings were reviewed for consistency and checked by a single researcher. Ten percent of the tracings were selected at random and traced by a second tracer, to confirm and maintain our high inter-rater reliability. Since our study relied on the color tracings of the PCSVN instead of the original raw images, the slight variation in the image acquisition process should pose little concern to the validity of our results as long as the images were clear enough for the tracer to identify the location of the vessels.

Color tracings were uniformly scaled and converted to 1380×1440 pixel binary images so the width of the vessels were normalized. One centimeter was marked with two blue dots on the ruler within the original photograph of the placenta to give scale. Roughly 35 pixels in the digital image corresponded to 1 cm on the placenta. Tracings were aligned so that the umbilical cord insertion lies at the center of the image. Each traced image was then fed through a series of MATLAB scripts, written completely by the researchers, to produce a fully connected graph network (e.g., Fig. 2 c) based on its color profile. Notice that, for example, in Fig. 2 c each branch point is marked with a solid dot to help with any calculation related to branch points. The 1-pixel-wide skeleton graphs were then used to produce 64 numerical values (e.g., Fig. 2 d), of which eight are shape-related (e.g., perimeter and area of placental chorionic surface plate) and 56 are vessel-related (e.g., number of branch points and vessel length).

Within the 56 vascular features, half of them were calculated on arterial networks and the other half were done on venous networks. Those features can be generally classified as counting descriptors (e.g., number of branches), measuring descriptors (e.g., arterial length), and relating descriptors (e.g., the distance between vessel and plate boundary). While similar analyses and results are available on the venous network, we will only present results on the arterial network here. The arterial networks are typically much more identifiable and visible than the venous networks. This allows the tracer to trace the arterial networks with a much higher level of precision and accuracy [14].

### Boruta algorithm for relevant feature selection

The Boruta algorithm is a feature selection algorithm for finding a minimal set of relevant variables. This method, which builds around the concept of random forest and decision trees [19], systematically and iteratively removes features that are less relevant than random probes by a statistical test [20–22]. By adding randomness to the system and collecting results from the ensemble of randomized samples one can reduce the misleading impact of random fluctuations and correlations and reduce the undesirable effect of over-fitting.

In the Boruta algorithm, each attribute has a “shadow attribute” which is created by shuffling the values of the original attribute. During a single run of Boruta, a feature attribute is deemed important if its importance score (*z*-score) is significantly bigger than the maximum *z*-score among all shadow attributes (MZSA). It is deemed unimportant if its *z*-score is significantly lower than the MZSA. A two-sided test of equality with the MZSA is performed on feature attributes that have undetermined importance. This process is repeated until the importance is assigned for all attributes or the algorithm has reached the previously set number of random forest runs, which is 500 in our simulations.

In conclusion of a Boruta simulation, a ranked list of features, ordered by their importance measure given in *z*-scores is produced. A major advantage of the Boruta strategy is its ability to discern truly important features from those that gain importance due to random correlations in data. Consequently, it gives us a powerful tool to establish a hierarchy of relevance when we need to study biological factors of various nature. In the current study, the Boruta algorithm allows us to confidently identify a list of PCSVN features that characterize the difference between high- and low-risk ASD placentas.

### Reduce dimensionality and collinearity with principal component analysis

Many features extracted from the Boruta algorithm remain correlated, making it harder to interpret the fundamental principles that govern villous growth. To this end, we used Principal Component Analysis (PCA) to reduce that collinearity, bringing a moderately large number of features down to a few independent signatures. These linearly independent components will be ranked by their proportion of contribution to the data variance. Precisely, if we let

$$F = \left[ \begin{array}{llll} \vert & \vert & & \vert \\ \mathbf{f}_{1} & \mathbf{f}_{2} & \cdots & \mathbf{f}_{p} \\ \vert & \vert & & \vert \end{array}\right] $$

be the *N*×*p* feature matrix, where *p*= number of samples (290 in this case) and *N*= the number of significant features selected by the Boruta algorithm, then the *N*×*N* covariance matrix $C = \frac 1{N-1} \tilde {F} \tilde {F}^{T}$ gives the feature variance on the diagonal entries and co-variance on the off-diagonal entries, where $\tilde {F}$ is centered around the feature mean. When $\tilde {F}$ is factored through its reduced singular value decomposition (SVD), $\tilde {F} = USV^{T}$, where *U*=[**u**_1_,**u**_2_,…,**u**_*k*_] is *N*×*k*, *S* is *k*×*k*, and *V* is *p*×*k*, the best feature basis (hence, the best feature space) to represent the data in the reduced *k*-dimensional space is stored in the *k* column vectors of *U* with *k*≪*N*. The best choice of *k* depends on how much variance we wish to capture. By representing the original data points through this new set of coordinates {**u**_1_,**u**_2_,…,**u**_*k*_}, the reduced-dimension data points, $D = U^{T} \tilde {F}$, are now expressed by a set of linearly independent principal components.

This allows us to investigate physical interpretations of these *N* features by finding which variables correlate most strongly with each component, i.e., finding which numbers are large in magnitude or the farthest away from zero in either positive or negative direction. Variables of large magnitude within the same principal component vary together, i.e., if one increases, then the remaining ones also increase. Thus, PCA was used to identify groups of biological effects of villous growth as a consequence of ASD risk.

### Classification with linear discriminant analysis

Associate each placenta in the data set with a *k*-dimensional vector, **p**, where each entry of **p** corresponds to a principal component coordinate. That is, **p** is a column vector in the matrix $D = U^{T} \tilde {F}$ in the previous section. To classify high-risk ASD placentas, Linear Discriminant Analysis (LDA) was conducted on the set of 290 placentas represented in the PCA coordinates. Suppose *D*_1_ and *D*_2_ are sets of PCA-reduced data points of low-risk and high-risk ASD placentas, respectively. Linear discriminant analysis amounts to finding a projection direction **w**_opt_ that maximizes the *between-class* scatter and minimizes the *within-class* scatter among the data points, which is equivalent to solving the optimization problem,

$$\mathbf{w}_{\text{opt}} = \text{argmax}_{||\mathbf{w}|| = 1} \frac{\left(\mathbf{w}^{T} \mathbf{m}_{2} - \mathbf{w}^{T} \mathbf{m}_{1}\right)^{2}}{S_{1}^{2} + S_{2}^{2}}, $$

where **m**_*i*_ is the *i*th class mean and $S_{i}^{2} = \sum _{\mathbf {y} \in D_{i}} \left (\mathbf {w}^{T} \mathbf {y} - \mathbf {w}^{T} \mathbf {m}_{i}\right)^{2}$ is the within-class scatter among the *i*th class.

The optimization problem is then solved through its matrix form: **w**_opt_=argmax *J*(**w**), where $J(\mathbf {w}) = \frac {\mathbf {w}^{T} S_{B} \mathbf {w}}{\mathbf {w}^{T} S_{W} \mathbf {w}} = \frac {N(\mathbf {w})}{D(\mathbf {w})}$. The between-class scatter matrix is given by *S*_*B*_=(**m**_2_−**m**_1_)(**m**_2_−**m**_1_)^*T*^ and the within-class scatter matrix is given by $S_{W} = \sum _{i=1,2} \sum _{\mathbf {x}\in D_{i}} (\mathbf {x}-\mathbf {m}_{i})(\mathbf {x}-\mathbf {m}_{i})^{T}$. **w**_opt_ is the largest eigenvector associated with the largest eigenvalue to the generalized eigen-problem *S*_*B*_ **w**=*λ* *S*_*W*_ **w**. This problem can be solved numerically through an SVD-based method.

Assume the projected values of the points in *D*_1_ fall to the left of those in *D*_2_. If we set the separation threshold, *α*, to be $\frac 12 \left (\min \left \{ \mathbf {w}_{\text {opt}}^{T} D_{2} \right \} + \max \left \{ \mathbf {w}_{\text {opt}}^{T} D_{1} \right \} \right)$, then a given placenta, **p**, is labeled low-risk for ASD if $\mathbf {w}_{\text {opt}}^{T} \mathbf {p} \leq \alpha $ and labeled high-risk for ASD if $\mathbf {w}_{\text {opt}}^{T} \mathbf {p} > \alpha $.

To generate error statistics, we perform LDA with a 10-fold cross validation. Essentially, the entire data set was randomly split into ten disjoint groups where each group of 29 placentas was used as testing probes to produce error statistics while the rest of the data set was used to find **w**_opt_ during each trial. Sample population (i.e., 30.69% of the population are high-risk for ASD and 69.31% are low-risk for ASD) was used in the model as an estimated priors to confirm that the use of Linear (instead of quadratic) Discriminant Analysis was the correct model.

## Results

### Feature selection and dimensionality reduction

**Fig. 3:**
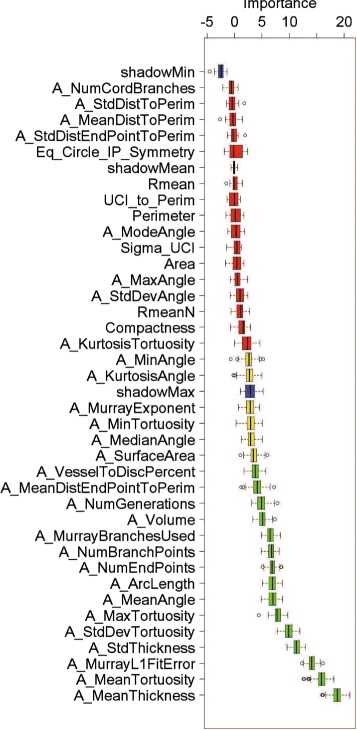
Feature selection result. Importance scores (horizontal axis) for each of the arterial vascular features (vertical axis) returned by the Boruta algorithm. A feature is considered the most relevant and ranked the highest when its importance score is the largest

The Boruta algorithm selected 15 arterial features. Figure 3 gives a visual output from running the Boruta algorithm in the programming language R. The box plot of each attribute, listed from the lowest (top) to the highest (bottom) rank, was the result of the *z*-score spread obtained from running the random forest algorithm 500 times. The 15 relevant and important features selected by Boruta are given in the “Vascular features” column of Table 1 and appear in green in Fig. 3.

PCA, implemented in MATLAB, was applied to the Boruta-selected 15-feature set. Five Principal Components (PCs) were retained to capture roughly 88% of the data variability. The principal components, also known as the eigenvectors of the covariance matrix, are given in Table 1 to delineate the source of contribution for each principal direction. Notice that many attributes within the same principal component are correlated. Next, we examine closely on the mathematical relationships of these features and deduce a list of independent principal features that will explain the biological and structural difference between the two cohorts. Here, we chose to borrow the term “principal” from “principal component” to describe independent features that are linear combinations of many other features.

#### Principal feature 1 – branch points

**Fig. 4:**
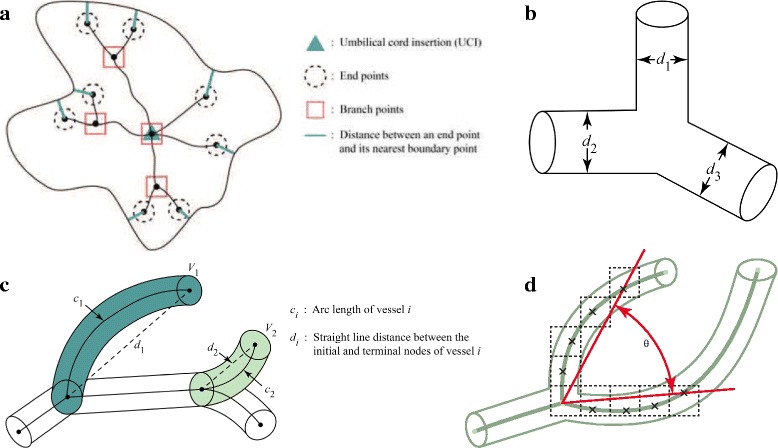
Visual definitions for the five principal features. **a** Principal feature 1 and 4: branch points and distance from an end point to its nearest point on the boundary. **b** Principal feature 2: vessel thickness, which is the same as the diameter (*d*_*i*_) of the *i* ^th^ vessel tube. **c** Principal feature 3: tortuosity, which is the ratio of arc length and straight line distance between the initial and terminal nodes of the vessel segment, i.e., tortuosity of the *i* ^th^ vessel = *c*_*i*_/*d*_*i*_. **d** Principal feature 5: branch angle, which is given by the angle between the two line segments formed by connecting the initial node and the fourth pixel of each branch

Each vascular network can be modeled by a mathematical tree, known as an undirected graph. There are two types of nodes on a vascular network – branch node and end node. As depicted in Fig. 4 a, a branch node is where a vessel splits into multiple branches and an end point/node is a terminal point on the network.

Let *x*=*NumEndPoints* be the total number of end nodes on the vascular network, *y*=*NumBranchPoints* be the total number of branch points found in the network, and *z*=*MurrayBranchesUsed* be the total number of branches that have child branches. Then *z* can be obtained by taking the difference between the total number of branches and *x*. If we further let *n*= number of nodes in the network, then the total number of branches is *n*−1. Overall, we have *n*=*x*+*y*. Therefore,

$$x = n - y \quad \text{and} \quad z = n - 1 - x = n - 1 - (n - y) = y - 1. $$

This is to say, all three vascular attributes with significant weights in PC1 are functions of *y*, *NumBranchPoints*.

**Table 1:** The first five principal components (PCs) of the data retain approximately 88% of the data variability

Boruta ranking	Vascular features (variability captured)	PC1 (35.27*%*)	PC2 (22.57*%*)	PC3 (17.20*%*)	PC4 (7.79*%*)	PC5 (5.80*%*)
1	MeanThickness	− 0.1582	− 0.4747	0.1035	0.0651	− 0.0089
2	MeanTortuosity	0.0002	0.0575	0.5347	− 0.0979	0.0013
3	MurrayL1FitError	− 0.256	− 0.3903	0.0438	0.0139	0.0397
4	StdThickness	− 0.1566	− 0.4762	0.0701	− 0.0046	0.0196
5	StdDevTortuosity	0.0029	0.0812	0.5912	− 0.0641	0.1449
6	MaxTortuosity	0.0948	0.0724	0.5459	− 0.0264	0.1709
7	MeanAngle	− 0.0611	0.0704	0.2028	0.2135	− 0.936
8	NumEndPoints	0.4251	− 0.0298	− 0.0132	0.0153	− 0.005
9	ArcLength	0.3773	− 0.1259	− 0.0035	− 0.0163	0.0116
10	NumBranchPoints	0.4254	− 0.0301	− 0.0125	0.0146	− 0.0038
11	MurrayBranchesUsed	0.4254	− 0.0301	− 0.0125	0.0146	− 0.0038
12	Volume	0.1444	− 0.4823	0.065	0.0502	− 0.0368
13	NumGenerations	0.3182	− 0.0237	0.014	0.2178	− 0.0619
14	MeanDistEndPointToPerim	0.0055	− 0.0323	0.0545	0.905	0.2124
15	VesselToDiscPercent	0.255	− 0.3502	0.0031	− 0.2561	− 0.1457

The absolute value of the attributes within each PC gives a measure of contribution. The higher the value, the bigger the contribution. Specifically, *NumEndPoints*, *NumBranchPoints*, and *MurrayBranchesUsed* contributed the most to PC1, *Thickness*, *StdThickness*, and *Volume* contributed the most to PC2, *MeanTortuosity*, *StdDevTortuosity*, *MaxTortuosity* contributed the most to PC3, *MeanDistEndPointToPerim* contributed most to PC4, and *MeanAngle* contributed most to PC5

#### Principal feature 2 – diameter/thickness

Vascular networks are intrinsically 3-dimensional tubular structure that can be modeled by circular cylinders. In this study, the diameter, which is a 3-dimensional feature of the vascular tubes, is treated the same as the width/thickness of the rectangular region obtained when tubes are pressed down, as depicted in Fig. 4 b. The “pressed-down” effect is similar to that of a stereographic projection. With this in mind, *MeanThickness* measures the average thickness among all arteries, i.e.,

$$MeanThickness = \frac1T \sum_{i=1}^{T} d_{i}, $$

where *d*_*i*_ is the thickness of the *i* ^th^ arterial vessel and *T* is the total number of arterial vessels exhibited in a single placental arterial network. *StdThickness* measures the standard deviation of thickness among all arterial vessels, i.e.,

$$ StdThickness = \sqrt{\frac1T \sum_{i=1}^{T} \left(d_{i}-\bar{d}\right)^{2}}, $$

where $\bar {d}$ is the *MeanThickness*. *Volume* gives the sum of all arterial volumes, i.e.,

$$Volume = \sum_{i=1}^{T} \pi\left(\frac{d_{i}}{2}\right)^{2} \cdot c_{i}, $$

where *c*_*i*_ is the arc length of the *i* ^th^ artery. All three features are functions of individual artery thickness and independent from the first principal feature.

#### Principal feature 3 – tortuosity

Tortuosity is a measure for the amount of twist or turns a curve has. It can be defined, in its simplest form, as the ratio of the length of the curve (*c*) to the distance between the ends of it (*d*), i.e.,

$$Tortuosity \, \text{of the} \, i^{\text{th}} \,\text{vessel} = \frac{c_{i}}{d_{i}}, $$

as depicted in Fig. 4 c. Severe tortuosity in vasculature can lead to various serious symptoms [23]. For example, tortuous artery and veins have been linked to aging, atherosclerosis, hypertension, genetic defects and diabetes mellitus in clinical settings [24–28].

With this definition, *MeanTortuosity*, *StdDevTortuosity*, and *MaxTortuosity* give the mean, standard deviation, and maximum of the arterial tortuosities. All three variables are estimators of network’s tortuosity which is independent from the number of branch points the network has and vessel thickness.

#### Principal feature 4 – growth extension

The bolded lines in Fig. 4 a illustrates the way we define the distance from an end point of the arterial network to its nearest point on the chorionic plate boundary. *MeanDistEndPtToPerim* represents the average distance between end points and their nearest point on the placental chorionic surface boundary, i.e.,

$$MeanDistEndPtToPerim = \frac1m \sum_{i=1}^{m} d_{i}, $$

where *m* is the total number of end points in the arterial network and

$$d_{i} = \min_{y \in \Omega} \|x_{i} - y\| $$

is the distance between each arterial end node, *x*_*i*_, and the nearest point *y* in the boundary curve, *Ω*.

*MeanDistEndPtToPerim* gives a notion of growth extension; that is, the smaller this value is, the more extended the network is to its boundary, on average. This measure is clearly distinct from principal features 1, 2, and 3 since there is no way we can calculate this value based on existing knowledge of the previous three.

#### Principal feature 5 – branch angle

Branch angles are used to capture the *instantaneous* growth at each branch point. For simplicity, we only consider vessels that bifurcate at their respective branch point, which make up more than 90% of the data. As illustrated in Fig. 4 d, branch angle is calculated as the angle between line segments that are formed between the branch point and the fourth pixel on the respective branch. The choice of four is an empirical decision to mimic the effect of instantaneous change. *MeanAngle* gives the average of all arterial vessels’ branch angles and does not depend on any of the previous four principal features. An alternative and popular approach to calculate branching angle is the one that finds the angle between two line segments joining the branch node and the end node. With this definition, branches that start off far apart but end up colliding at a single node would have an angle of 0. This alternative notion of the branching angle does not accurately capture the instantaneous growth behavior at branch points; hence, not ideal in our analysis.

### Visualization of high- and low-risk ASD cohorts

**Fig. 5:**
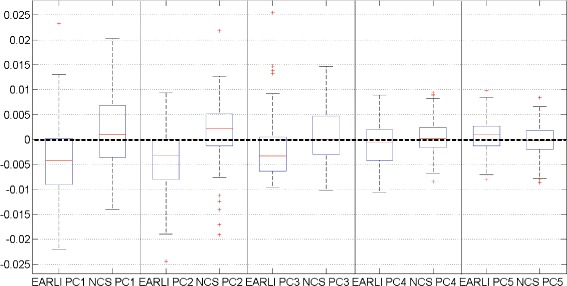
Visible difference between high- and low-risk ASD groups in low dimensions. The box whisper plot of the projection coefficients for the first five principal components of EARLI (89 data points) and NCS (201 data points) cohorts. The difference between the two groups are apparent and consistent across all five PCs. For example, the mean of the first PC projection coefficients among the EARLI placentas is negative while the mean of the first PC projection coefficients among the NCS placentas is positive

**Fig. 6:**
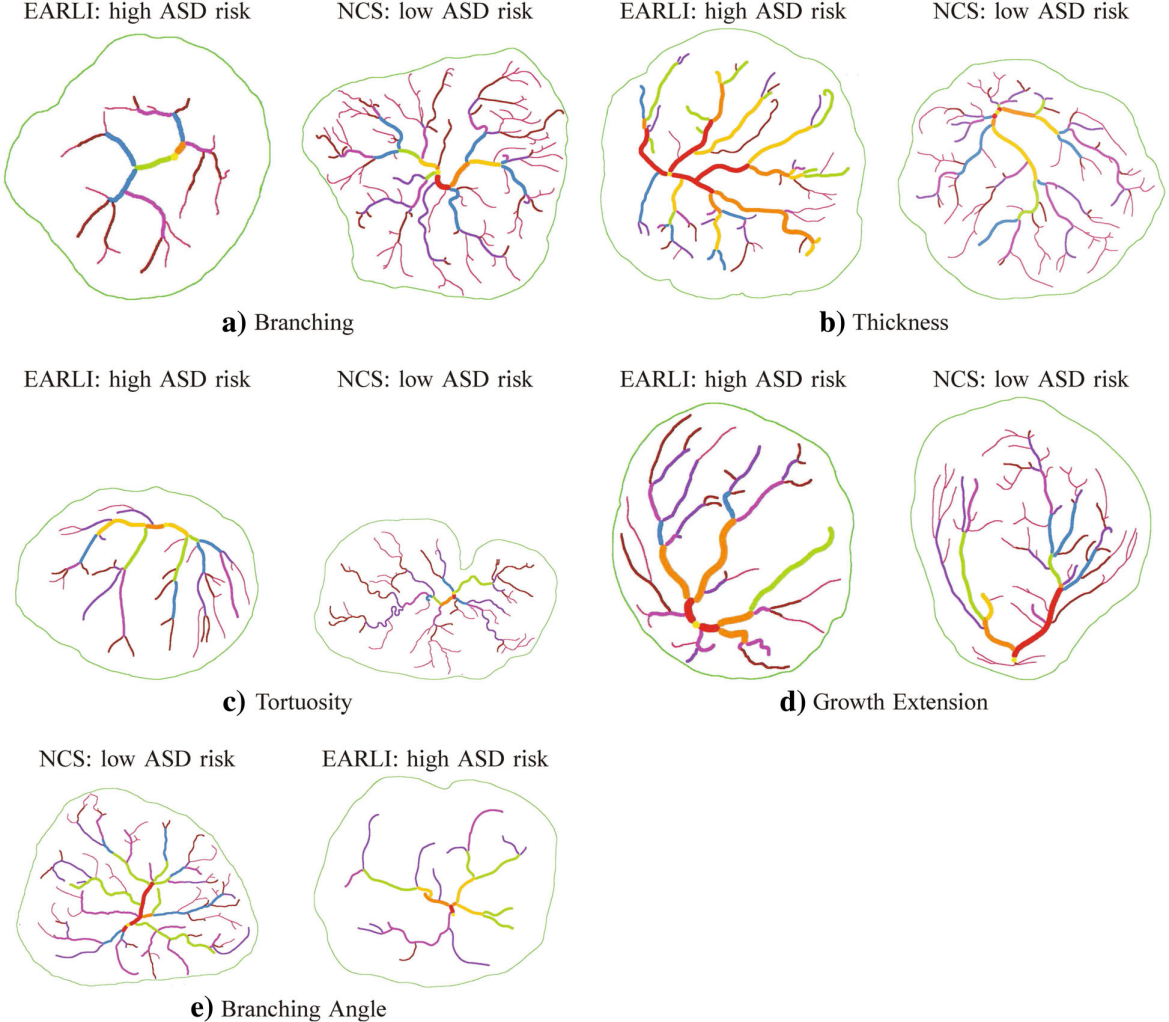
Images with the lowest (left) and the highest (right) Principal Component (PC) projection coefficients for the first five PCs. **a** Principal feature 1: number of branch points. For example, the average number of branch points is 36.74 with a standard deviation of 15.66 in EARLI (left) and 48.48 with a standard deviation of 16.34 in NCS (right). **b** Principal feature 2: thickness. For example, the average mean thickness is 0.16 with a standard deviation of 0.03 in EARLI (left) and 0.13 with a standard deviation of 0.02 in NCS (right). **c** Principal feature 3: tortuosity. For example, the average standard deviation of tortuosity is 0.06 with a standard deviation of 0.03 in EARLI (left) and 0.08 with a standard deviation of 0.03 in NCS (right). **d** Principal feature 4: growth extension. For example, the average mean distance from end points to the nearest boundary point is 2.82 with a standard deviation of 0.48 in EARLI (left) and 2.96 with a standard deviation of 0.41 in NCS (right). **e** Principal feature 5: branching angle. For example, the average mean branching angle is 102.28 with a standard deviation of 2.87 in NCS (left) and 100.64 with a standard deviation of 3.51 in EARLI (right)

The numerical distribution of the high- and low-risk ASD placentas in each of the five principal features can be seen in Fig. 5. The difference between the two groups were particularly pronounced in the number of branch points, vessel thickness, and vessel tortuosity. These differences can be visualized more clearly when we compare the most extreme cases within each principal feature, as illustrated in Fig. 6. For example, the average number of branch points in the EARLI placentas was a lot lower than that in NCS, as illustrated by Fig. 6 a. Similarly, a significant difference between the two groups was found in each of the other four principal features as shown in Fig. 6.

### Classification result of the high-risk ASD placentas

LDA with a 10-fold cross validation (CV), implemented in MATLAB, was performed to examine how well the selected principal features work together to classify placentas with increased ASD risk. The average error rates across all 10 validation trials were 6.90*%* and 8.97*%* for false positives and false negatives, respectively. The results suggested that on average, we were able to correctly tell whether a given placenta belongs to a low-risk or high-risk ASD cohort 84 out of 100 times based on various constructs of the five extracted principal arterial features. Among the ones missed, roughly 9% were EARLI placentas misclassified as NCS placentas and 7% were NCS placentas misclassified as EARLI placentas.

To increase the reliability of our results, we additionally implemented a stratified 10-fold cross validation to take into consideration of the class imbalance in the data set. The result was comparable to our original CV result with an overall misclassification of 15.12%.

## Discussion

A major contribution of our work is the creation and validation of a model to classify placentas associated with children in a high-risk ASD group against a population of unknown ASD risk based on automatically selected PCSVN features. The feature-selection algorithm that is based on the Boruta method returned 15 ranked attributes in an ensemble of 28 arterial features and 8 shape-related features. The fact that the Boruta method ranked arterial features higher than all of the shape-related features tells us that the difference in ASD risk can be explained by arterial features alone. We benchmarked our results with another feature selection method called Elastic Net [29], which is also known to minimize over-fitting issues. The Elastic Net method returned a set of 16 features. Among the 16 features, 14 were identical to the Boruta result with two new features, *MedianAngle* and *KurtosisTortuosity*. The feature *VesselToDiskPercent* was present in the Boruta method only. We then conducted a Principal Component Analysis on the set and noticed that the principal features selected were, sorted by the amount of variance captured, (1) number of branch points, (2) tortuosity, (3) thickness, (4) branching angle, and (5) growth extension. Notice that the types of principal features selected by the Elastic Net and PCA combination are identical to those selected from the Boruta and PCA combination. The only difference is in the amount of the variability each feature captures.

**Fig. 7:**
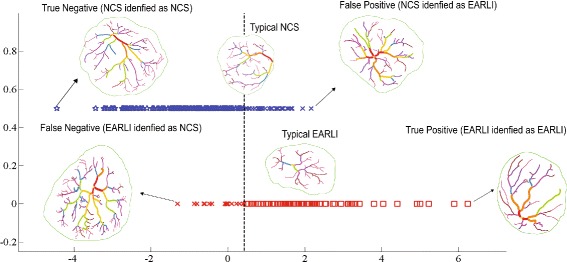
A visualization of the Linear Discriminant Analysis (LDA) result. Each placenta in the data set is associated with a dot in the projected space. The vertical dashed line serves as a separation threshold. In the case of a perfect separation, all points on the top line should fall to the left of the threshold while all points on the bottom line should fall to the right of the threshold. The graph illustrates cases for which the classifier has an easier time (True Negative and True Positive) and a harder time (False Positive and False Negative) predicting

The statistical significance of our results was established through the Linear Discriminant Analysis with a 10-fold cross validation. Specifically, our classifier trained on the five PCA-reduced principal features placed unlabeled placentas in the correct group nearly 84% of the time. We were able to improve the overall classification rate to slightly above 90% with a non-linear classifier called support vector machine (SVM). As mentioned earlier, since NCS was population-based, one would expect that some small number of the pregnancies resulted in a child with ASD and would thus have been “high-risk.” Therefore, a perfect classification result was unlikely. The improved classification rate afforded by SVM might therefore be a result of over-fitting. The misclassification result returned by LDA can be visualized in Fig. 7. Specifically, when a high-risk ASD placenta had too many branch points, thinner and tortuous arterial vessels, larger branching angles, and did not extend closely to the surface boundary, it was treated as if it were a low-risk ASD placenta.

Research [7] has shown that about 20% of the high-risk group will go on to have ASD, compared to roughly 1.5% in the low-risk group. Among the ASD high-risk group, 30–40% will have other developmental delays, compared to roughly 5–15% in the low-risk group. That is, the remaining 40–50% of the ASD high-risk placentas will be typically developing. For this reason, one should expect that the collections of PCSVN signatures selected by Boruta and PCA for the high-risk ASD and the diagnosed ASD placentas will not overlap completely. We made no attempt to differentiate the vascular features of the placentas associated with ASD and those associated with other developmental delays since our data does not come with such diagnoses. Our study offers no additional insight into what could have caused the placentas associated with the high ASD risk to grow this way. That is, it remains unclear what PCSVN characteristics is unique to ASD placentas.

Some interesting questions to ask next include what environmental or genetic factors cause this group of five parameters to vary together and whether these variables stabilize in their permanent state early in gestation. Furthermore, searching for the types of geometric signatures that are measurable and capable of providing accurate readings in 3-dimensional imaging environment is also going to play a vital role in early risk assessment and intervention for ASD.

Many important ultimate placental morphologic features are likely predetermined early in pregnancy. Reliable quantization of PCSVN features will provide researchers useful tools to study the intrauterine origins of diverse disease outcomes and lead to the development of methods more broadly applicable to other branched structures including other vascular networks. Improved understanding of the details of early placental development as expressed in PCSVN branching morphogenesis may shed light on the interplay between the fetal genetic program and intrauterine environmental factors that may vary across gestation [30, 31]. Because the placenta is key to the development of many fetal/perinatal/neonatal and potentially lifelong health risks, the work presented here helps to translate placental research that can clarify timing and nature of conceptus compromise into potentially actionable clinical risk assessment.

The study presented here should motivate a pursuit of additional PCSVN features which might be correlated with various dichotomous health outcomes as long as information on outcome classification is available. We anticipate that some PCSVN features will correlate with outcomes such as diabetes, obesity, hypertension and cardiovascular disease or other “fetal origins” disorders, including autism and schizophrenia, once reliable and automated vessel extraction methods are established to allow analysis of PCSVNs in large cohorts.

Since digital images of PCSVN can be captured and analyzed within days of delivery, our classification model allows us to determine, within minutes, which risk group a new placenta belongs to. This information can be one of the multiple measures doctors use to make recommendations for early ASD interventions in clinical settings. However, a major barrier in implementing the results of our work in clinical practices is the availability of trained human tracers. Tracing PCSVN is the most time-consuming and laborious step in the entire classification pipeline. Researchers are currently developing reliable methods to automatically extract placental vascular networks from digital images of placental chorionic surface [32, 33] in order to bypass the need for manually traced images.

## Conclusions

Our study specifically demonstrated that the arterial networks that are associated with a high risk for ASD tend to have a fewer number of branch points, thicker and less tortuous vessels, better extension to the surface boundary, and smaller branch angles than their population-based counterparts. These five independent geometric features work collectively to provide a discriminating vascular signature for the high-risk ASD placentas. This result does not imply that all high-risk ASD children will have placentas satisfying each of those five conditions simultaneously; rather, these features, when taken as a whole, provide substantive discriminatory power.

The combination of the feature-selection and classification algorithms presented herein provides a mechanism in discriminating placentas from high-risk ASD pregnancies against those from a population-based cohort with unknown risks based on automatically selected PCSVN features. Although our study which built upon a single risk cohort can only offer limited implications, our work is readily transferrable to studying other adult and neonatal diseases. We will be in a great position to conduct a comprehensive study across many disease cohorts as soon as the data becomes available.

## Abbreviations

ASD: Autism spectrum disorder
EARLI: Early autism risk longitudinal investigation
LDA: Linear discriminant analysis
MZSA: Maximum *z*-score among all shadow attributes
NCS: National children’s study
PCA: Principal component analysis
PC: Principal component
PCSVN: Placental chorionic surface vascular network
SVM: Support vector machine
